# Usefulness and Usability of a Personal Health Record and Survivorship Care Plan for Colorectal Cancer Survivors: Survey Study

**DOI:** 10.2196/10692

**Published:** 2019-08-20

**Authors:** Will L Tarver, Bruce W Robb, David A Haggstrom

**Affiliations:** 1 VA Health Services Research and Development Center for Health Information & Communication Richard L Roudebush VA Medical Center Indianapolis, IN United States; 2 Department of Health Policy & Management Fairbanks School of Public Health Indiana University Indianapolis, IN United States; 3 Department of Surgery School of Medicine Indiana University Indianapolis, IN United States; 4 Division of General Internal Medicine and Geriatrics School of Medicine Indiana University Indianapolis, IN United States; 5 Center for Health Services Research Regenstrief Institute Indianapolis, IN United States

**Keywords:** personal health record, colorectal cancer, survivorship, digital health, digital medicine

## Abstract

**Background:**

As a result of improvements in cancer screening, treatment, and supportive care, nearly two-thirds of individuals diagnosed with colorectal cancer (CRC) live for 5 years after diagnosis. An ever-increasing population of CRC survivors creates a need for effective survivorship care to help manage and mitigate the impact of CRC and its treatment. Personal health records (PHRs) and survivorship care plans provide a means of supporting the long-term care of cancer survivors.

**Objective:**

The purpose of this study is to characterize the usefulness of a CRC PHR and survivorship care plan and to describe the usability of these technologies in a population of CRC survivors. To our knowledge, this is the first study to assess a PHR and survivorship care plan specifically targeting CRC survivors.

**Methods:**

Twenty-two patients with CRC were recruited from surgery clinics of an academic medical center and Veterans Affairs hospital in Indianapolis and provided access to an online Colorectal Cancer Survivor’s Personal Health Record (CRCS-PHR). Survey data were collected to characterize the usefulness of the CRCS-PHR and describe its usability in a population of CRC survivors. CRC survivors were surveyed 6 months after being provided online access. Means and proportions were used to describe the usefulness and ease of using the CRC website. Open-ended questions were qualitatively coded using the constant comparative method.

**Results:**

CRC survivors perceived features related to their health care (ie, summary of cancer treatment history, follow-up care schedule, description of side effects, and list of community resources) to be more useful than communication features (ie, creating online relationships with family members or caregivers, communicating with doctor, and secure messages). CRC survivors typically described utilizing traditional channels (eg, via telephone or in person) to communicate with their health care provider. Participants had overall positive perceptions with respect to ease of use and overall satisfaction. Major challenges experienced by participants included barriers to system log-in, lack of computer literacy or experience, and difficulty entering their patient information.

**Conclusions:**

For CRC, survivors may find the greater value in a PHR’s medical content than the communication functions, which they have available elsewhere. These findings regarding the usefulness and usability of a PHR for the management of CRC survivorship provide valuable insights into how best to tailor these technologies to patients’ needs. These findings can inform future design and development of PHRs for purposes of both cancer and chronic disease management.

## Introduction

In 2016, almost 1.5 million people in the United States were expected to be living with a history of colorectal cancer (CRC) [1]. Although CRC continues to be the third most common cancer among both men and women [2], improvements in cancer screening, treatment, and supportive care have led to decreases in cancer mortality rates [3-5]. As a result, nearly two-thirds of the individuals diagnosed with CRC live for 5 years after diagnosis [6]. An ever-increasing population of CRC survivors creates a need for effective survivorship care to help manage and mitigate the impact of CRC and its treatment. Although the reduction in cancer mortality can be partially attributed to cancer treatments, many of the same treatments carry substantial risks and expose patients to adverse long-term or late effects [7]. In addition, up to 40% of CRC survivors develop recurrent disease [8], a fact that also leads to cancer worry among survivors [9]. Therefore, CRC survivorship care should include the identification and management of physical and psychological effects of CRC treatment, surveillance for cancer recurrence, and improved communication with providers [10] in order to fully address the needs of this population.

The use of health information technologies has been identified as a means of supporting the long-term care of cancer survivors [11]. However, there is a lack of evidence supporting patient-centered technologies including personal health records (PHRs) for this purpose [12,13]. This finding may result from little or no emphasis on the acceptability and usability of these technologies to the patients using them and the barriers to successful implementation of PHRs [14,15]. Common barriers to the optimal use of PHRs include the negative attitudes of patients (eg, perceiving self-tracking as extra work) and providers (eg, seeing the PHR as extra work), interface challenges, and privacy concerns [16]. Patient-centered technologies that undergo usability testing have been found to have greater success in overcoming barriers and achieving positive outcomes [16]. Existing literature on PHR usability in cancer care has been largely limited to breast cancer and shown positive results when these technologies are tailored to the needs of patients [17,18]. Jacobs and colleagues sought to understand the usability of a health management aid and found that effective use was associated with the development of a tool that was customizable, mobile, and integrated into the care of patients [18]. In the case of a clinical trial matching system embedded in a Web-based PHR, Atkinson and colleagues found that changing content and attending to usability issues improved breast cancer patients’ satisfaction with the technology [17]. Thus, such approaches may prove valuable for improving the impact of PHRs for CRC survivors.

Although the literature on the use of cancer-specific PHRs focuses on breast cancer, the usefulness of these Web-based technologies may vary by the type of cancer. Every cancer type is unique in its patient needs, treatment approach, and follow-up strategy. For example, a common side effect of breast cancer treatment is lymphedema, or swelling of the arms. Conversely, a common side effect of CRC is the need for an ostomy bag. Both represent challenges a patient must manage, which may be aided by an appropriately tailored technology. With respect to individual cancers, the usefulness of an online technology cannot be taken for granted. Importantly, the perspectives of the end user (patients with CRC) are vital to develop a patient-centered PHR tailored to the needs of the end user [19]. The purposes of this study are to characterize the usefulness of a CRC PHR and survivorship care plan and to describe the usability of this CRC PHR and survivorship care plan among a population of CRC survivors. To our knowledge, this is the first study to assess a PHR and survivorship care plan specifically targeting CRC survivors.

## Methods

### The Colorectal Cancer Survivor’s Personal Health Record

The Colorectal Cancer Survivor’s Personal Health Record (CRCS-PHR) was developed by adapting an open-source electronic health record (OpenMRS) [20] to deliver an online survivorship care plan to CRC survivors. The chosen features of the CRCS-PHR were drawn from an Institute of Medicine report, which recommended that every cancer patient receive a survivorship care plan summarizing information important to the individual’s long-term care [21]. This information includes a treatment summary, type of cancer and treatments, and a survivorship care plan consisting of potential side effects of treatment and specific information about the recommended follow-up (surveillance) care. In the development of the CRCS-PHR, the guiding principle when making design decisions was patient centeredness; consistent with this approach, we created a technology to make medical information accessible to the patient, empower the patient to manage information through decision-support tools, and allow the patient to control whom the information would be shared with. Table 1 summarizes the functions of the CRCS-PHR (see Multimedia Appendix 1 for further details).

Users could create online relationships with their doctors of choice, whether primary care or specialist physicians. Participants did not need to download any particular software to use the Web-based CRCS-PHR. Given the information complexity of certain functions of the CRCS-PHR, the system had not yet been designed for the smaller visual window of mobile devices. CRC survivors were instructed how to use the CRCS-PHR at the time of study recruitment in person at health care clinics. Subsequently, both a video tutorial and detailed user’s guide were available online to provide patients with directions on using the system.

**Table 1:** Description of functions of the Colorectal Cancer Survivor’s Personal Health Record.

Function	Description
Treatment summary	Summarizes cancer diagnosis and treatment, including type of surgery and adjuvant therapy (chemotherapy or radiotherapy)
Side effects	Tailored compendium of possible side effects of treatment
Surveillance care	Surveillance tests are recommended to detect cancer recurrence. Tailored reminders about guideline-concordant surveillance care were delivered to the patient. A table also summarized tests completed and the next test due.
Community resources	Links to cancer survivor information resources and support groups.
Relationships	Patients identify role-based individuals (provider, caregiver) with whom to share the personal health record. Relationships are configurable to allow access to part or all components of the personal health record.
Journal	Patients enter *unstructured* information about their experience with cancer and treatment into a journal (using My Relationships; others with whom the journal is shared can write in-line comments)
Secure messaging	Enables patients to send and receive messages from individuals with whom they have created a relationship.

The Treatment Summary section of the CRCS-PHR provided patients with access to detailed information about their cancer type and treatment received, including surgery, radiation therapy (type and duration), and chemotherapy (type and duration), as well as any complications associated with treatment. Name and contact information were also provided regarding the primary care and treating physician of all modalities (surgeon, radiation oncologist, medical oncologist, and primary care physician). However, the Web-based system did not connect directly with their institutional or vendor-based electronic health record.

The research team obtained information presented in the Treatment Summary from the electronic health record and entered it into the CRCS-PHR on behalf of the patient at the time of enrollment into the study. Possible side effects were automatically communicated to the patient in the CRCS-PHR with an algorithm based on the treatment received (surgery, chemotherapy, or radiation therapy). Similarly, surveillance care reminders were automatically delivered to the patient with an algorithm based on cancer diagnosis (colon or rectal) and stage (I-III). Information about completed surveillance care and results were self-entered by the patient.

We conducted a feasibility study of the CRCS-PHR to determine the perceived usefulness and usability of the targeted PHR intervention among CRC survivors. The goal of the study was to gather information to guide the iterative development of the CRCS-PHR.

### Study Sample

Recruitment sites included surgery clinics at an academic medical center and Veterans Affairs (VA) hospital in Indianapolis. To be eligible, patients with CRC had to have received curative-intent therapy and be diagnosed with stage I-III CRC between 2 months and 30 months prior. Participants were excluded if they had metastatic disease or did not speak English. A total of 22 cancer survivors were recruited; a minimum of 20 patients was considered an appropriate recruitment goal for this feasibility study. Data were collected to better understand the needs and experience of patient end users prior to conducting a large, randomized controlled trial. All participants were surveyed 6 months after being provided online access to the CRCS-PHR in order to assess its usefulness, ease of use, and overall satisfaction.

### Measures

#### Usefulness

Eleven items assessed the perceptions of usefulness patients associate with different elements of CRCS-PHR (scale of 1=not at all useful to 10=very useful): (1) Summary of my cancer treatment history, (2) Reviewing my follow-up care schedule, (3) Self-entering follow-up tests I had received, (4) Description of side effects, (5) List of community resources, (6) Creating and setting up relationships with family members or caregivers, (7) Communicating about my cancer diagnosis with family members or caregivers, (8) Creating and setting up a relationship with my doctor, (9) Communicating with my doctor, and (10) Sending mail messages through the cancer website.

#### Ease of Use and Overall Satisfaction

Five items assessed ease of using the CRCS-PHR (scale of 1=poor to 10=excellent): (1) Ease of reading the site, (2) Overall organization of information of the site, (3) Ease of navigating the tabs on the site, (4) Ability to find information you want on the site, and (5) How fast the pages appear after you click on the link. Three items assessed the overall satisfaction with the CRCS-PHR features (scale of 1=not at all to 10=very well): (1) How well did the cancer website meet your expectations? (2) How likely are you to recommend this website to other cancer survivors? (3) Considering all of your experiences to date, how satisfied are you with the cancer website overall? In addition, three open-ended questions were used to assess barriers and facilitators to CRCS-PHR use: (1) What were barriers (or things that made it hard) for you to use the cancer Website? (2) What were facilitators (or things that made it easy) for you to use the cancer website? (3) What is the main improvement that you would suggest for the cancer Website?

### Ethics Approval

The study procedures and protocol were approved by the Indiana University-Purdue University Institutional Review Board for the protection of human subjects and the VA Research and Development Committee.

### Statistical Analysis

Means and proportions were used to describe the study population, usefulness of the CRCS-PHR features, and ease of using the CRCS-PHR. All quantitative analyses were conducted using Stata statistical software (version 15.1; StataCorp, College Station, TX). Open-ended questions were qualitatively coded and analyzed by two coders working together using the constant comparative method [22]. This method involves reviewing the open-ended survey responses and then comparing them with the others that followed in order to identify themes based on the possible relations between each prior code [22]. Similar responses to each question were coded and grouped together.

## Results

### Overview

As seen in Table 2, slightly more than half of the participants were men (55%), which is comparable to the national CRC average of 52.7% [3]. The average age of participants with CRC in this study was 58 years, which is lower than the national average of approximately 70 years for patients with colon cancer and 63 years for patients with rectal cancer [3]. In addition, slightly more than half of the participants were college graduates or had a postgraduate degree (55%) or were employed full-time (54%). Most participants were married (68%) and earned at least US $50,000 annually (64%).

**Table 2:** Characteristics of the study sample (N=22).

Characteristic		Value
Age (years), mean (SD)		58 (9.50)
**Gender, n (%)**		
	Male	12 (55)
	Female	10 (45)
**Marital status, n (%)**		
	Married	15 (68)
	Other	7 (32)
**Education, n (%)**		
	Less than high school	0 (0)
	High school or General Education Development	6 (27)
	Some college or technical school	4 (18)
	College graduate or postgraduate degree	12 (55)
**Income (US $), n (%)**		
	<30,000	6 (27)
	30,000-50,000	2 (9)
	>50,000	14 (64)

### Usefulness of Colorectal Cancer Survivor’s Personal Health Record

CRC survivors’ perceptions of the usefulness of the CRCS-PHR are presented in Table 3. On average, survivors tended to perceive features related to their care to be useful (measured on a 10-point Likert scale, where 1=not at all useful and 10=very useful). The highest-rated medical care features were found to be the summary of the patient’s cancer treatment history and follow-up care schedule. However, self-entering follow-up tests was found to have slightly lower-than-average usefulness. In addition, overall, survivors tended to perceive features related to communication as not as useful.

### Ease of Use and Overall Satisfaction

Survivors’ perceptions of the usability of the CRCS-PHR are listed in Table 3. With regard to the ease of using the CRCS-PHR, participants had overall positive perceptions. However, participants were neutral with respect to how fast the pages appear after you click on the link. With regard to satisfaction, participants were overall satisfied with their use of the CRC-PHR.

Participants preferred to receive access to the CRCS-PHR when first diagnosed with CRC. With regard to the patients’ view of when they would prefer access to the cancer website, a majority of patients preferred to receive access “Right away, when [they were] first diagnosed with colorectal cancer” (n=17, 77%).

**Table 3:** Perceived usefulness, ease of use, and satisfaction with Colorectal Cancer Survivor’s Personal Health Record in the study sample (N=22).

Measures^a^		n (%)	Score, mean
**Usefulness of medical care features (1=not at all useful, 10=very useful)**			
	Summary of my cancer treatment history	19 (86)	6.4
	Reviewing my follow-up care schedule	20 (91)	6.3
	Self-entering follow-up tests I had received	20 (91)	4.9
	Description of side effects	20 (91)	5.7
	List of community resources	19 (86)	5.4
**Usefulness of communication features (1=not at all useful, 10=very useful)**			
	Creating and setting up relationships with family members or caregivers	20 (91)	4.1
	Communicating about my cancer diagnosis with family members or caregivers	20 (91)	3.8
	Creating and setting up a relationship with my doctor	20 (91)	4.6
	Communicating with my doctor	20 (91)	4.6
	Sending mail messages through the cancer website	20 (91)	4.3
**Ease of using the CRC^b^** **website (1=poor, 10=excellent)**			
	Ease of reading the site	20 (91)	7.7
	Overall organization of information of the site	20 (91)	7.1
	Ease of navigating the tabs on the site	20 (91)	7.2
	Ability to find information you want on the site	20 (91)	7.5
	How fast the pages appear after you click on a link	20 (91)	4.8
**Satisfaction with the CRC website (1=not at all, 10=very well)**			
	How well did the cancer website meet your expectations?	20 (91)	6.2
	How likely are you to recommend this website to other cancer survivors?	21 (95)	7.6
	Considering all of your experiences to date, how satisfied are you with the cancer website overall	20 (91)	6.3
**Preference of timing to receive access to the cancer website: If given the chance, when would you first like to have had access to this cancer website?**			
	Right away, when I was first diagnosed with colorectal cancer	17 (77)	N/A^c^
	Not right away, but before any treatment for cancer	2 (9)	N/A
	After surgery, but before other treatments	1 (5)	N/A
	During treatment (including radiation or chemotherapy)	0 (0)	N/A
	After all treatment is completed (including radiation or chemotherapy)	1 (5)	N/A

^a^All responses to individual survey items were included. One respondent only answered the question “How likely are you to recommend this website to other cancer survivors?” and another respondent did not rate the usefulness of all medical care features.

^b^CRC: colorectal cancer.

^c^N/A: not applicable.

### Open-Ended Responses

Table 4 presents representative examples from the answers to open-ended questions that reflect recurrent themes within the qualitative data that were expressed by more than one participant. The major challenge experienced by participants was logging into the system. Other challenges included inexperience and lack of computer literacy as well as difficulty entering their patient information. Facilitators to use of the CRCS-PHR included a user-friendly interface and easy navigation.

Participants stated that the CRCS-PHR was most valuable with respect to its medical care functions; however, it would have been more useful earlier in their cancer journey. With respect to communication, participants typically described resorting to traditional means of communication with their health care provider (ie, in person or via telephone). Participants also expressed interest in communicating with other CRC survivors in online networks in order to have a support group of individuals who had similar experiences.

**Table 4:** Representative responses from the open-ended questions.

Theme	Excerpt
Barriers to use	Logging in - could use it at first and then couldn’t use it Getting password and making part of routine Password - could not change it Lack of computer skills Inputting my own information because it was time-consuming
Facilitators to use	Self-explanatory & navigate tabs, very user friendly Easy to understand and find information Didn’t have to think much (user friendly) Easy to navigate
Communication with providers	Rather talk in person Easier to contact over phone Lack of time & rather talk in person
Communication with family, caregivers, and friends	More of a private person Private about medical information Like to keep things private
Communication with other CRC^a^ survivors (suggested improvements)	More exchange to other cancer survivors Website for specific cancers for others with same cancer to network Highlight resources more with specific feature - CRC networking site

^a^CRC: colorectal cancer.

## Discussion

### Principal Findings

We found divergence between the perceived usefulness of medical care functions compared to communication functions. Participants reported that the majority of medical care functions of the CRCS-PHR had better than average usefulness. This finding is consistent with a qualitative study of CRC patients and providers, which found that CRC patients wanted to have general and tumor-specific health information and be able to track the course of illness and treatment over time [19]. Conversely, participants found communication functions less useful. Although patients are interested in communicating with their providers electronically [23,24], older individuals are less likely to communicate online with a health care provider. Our qualitative, open-ended responses provided further insight into why participants might have given communication functions lower scores. Communication functions, from the patient perspective, may be better handled by other platforms such as via the telephone or other nonelectronic modes of communication.

Given that our participants reported limited experience using technology, they may resort to forms of communication with which they are more familiar when communicating with their health care provider. Several participants mentioned that it was easier to call their doctors than to communicate with them electronically. This is consistent with another study that found that patients viewed communication through the PHR as cumbersome and preferred contacting their provider’s office directly [25]. Although another study found that patients viewed direct communication with their providers as a valuable feature, the lack of computer proficiency was cited as a barrier to using PHRs [26]. A previous review found that patients and providers were more likely to find these functions useful if they perceived them to be more beneficial than the existing options [16]. Successful use is also dependent on the buy in from providers who assure their patients that this form of communication is meant to supplement the existing patient-provider relationship, not replace it. Factors limiting provider buy in include provider perceptions that the PHR will result in extra work being added to their current clinical responsibilities [16] as well as concerns that patients will perceive them as being permanently on call [19].

Divergence between the perceived usefulness of medical care and communication functions may also be explained by several other factors. Patients may view medical data as information that is uniquely held by health care providers. Consequently, an online portal that provides tools for patients to obtain this previously inaccessible information may be considered to have great value. Conversely, online tools that facilitate communication with family members or caregivers may provide a solution to an issue that patients do not perceive as a problem. Cancer survivors may also be more reluctant to communicate with their providers online than the general population due to the personal nature of their disease or heightened concerns about privacy.

Participants reported that they would have preferred to receive the intervention either when first diagnosed or before treatment; many perceived that they received the intervention too late to receive the full benefits. Previously, concerns have been expressed about information overload at the time of diagnosis and that patients may have a difficult time remembering or processing information initially shared due to stress. However, data from this study suggest that patients are receptive to receiving survivorship care plans earlier, which indicates that they are aware of the importance of information about cancer follow-up, enabling them to plan ahead [27].

Participants reported mixed experiences with respect to the ease of use of the CRCS-PHR. Although participants overall responded favorably to the interface, several reported issues with logging into the system. Participants were assigned passwords and able to communicate with the research team to have their password changed. Feedback from participants suggests that allowing them to select their own password and change passwords in an automated manner may remove the obstacles to accessing the CRCS-PHR. Initial access to patient portals and login problems have been a commonly observed problem [28,29]. Additionally, survivors flagged issues related to the downloading time for the CRCS-PHR. Slow download speeds highlight another dimension of access, and rural populations may be especially vulnerable, living in communities that lack high-speed broadband access.

Participants expressed concern about the amount of information they needed to input into the CRCS-PHR such as information about provider visits and treatments. Although the literature suggests that patients can reliably enter information for systems, including easy-to-measure biometrics such as height, weight, and temperature, most patients are unable to reliably report specific laboratory values [30]. When implementing a cancer survivorship care plan, PHRs can be tethered to health care providers’ electronic health record, so that medical information is automatically transferred from the electronic health record to the PHR. Such processes would both minimize patient data entry and improve data accuracy, making the CRCS-PHR platform more scalable.

Similar to other studies reporting limitations related to the use of PHRs [25,26,31,32], some participants acknowledged a lack of experience using computers. Providing participants with access to basic training on the use of computers when needed would facilitate the use of these technologies. Short training sessions have been found to reduce computer anxiety and increase computer interest and self-efficacy among older adults [33,34].

### Limitations

Our study recruited clinic-based samples from academic and VA health care settings, and thus, our findings may not be fully generalizable to cancer survivors seen in other community health care settings. The population was largely Caucasian, and experiences may be different among other racial or ethnic groups. Further, the mean age of the population (58 years) was lower than the average age of CRC patients (70 years). The use of new technologies may be easier among relatively younger patients; however, as the digitally proficient population ages, the use of online technologies will become more widespread. In addition, the developers and evaluators were separate teams managed by a common leadership (DH, principal investigator), and this organizational structure may have biased the study findings in favor of the CRCS-PHR; however, our study measures and analyses were prespecified, thereby limiting the influence of any unconscious bias. Finally, the study’s cross-sectional design did not allow us to ascertain whether the perceived usefulness or usability of the tool changed over time with continued use.

### Conclusions

Survivors highlighted potential opportunities for the PHR to provide additional value in supporting their cancer care. This report is the first published study on the usability and usefulness of a PHR for the management of CRC and provides valuable insight on tailoring these technologies to patients’ experiences. For CRC, patients may find the greater value in a PHR’s medical care content than its communication functions, which are available elsewhere. Despite concerns about information overload, patients clearly expressed a preference to receive their care plan closer to the time of diagnosis and before the onset of treatment rather than later in the cancer care continuum. Like providers, patients may find data entry burdensome. Tethering these technologies to existing electronic health records would reduce this burden. Taken together, these findings will inform future redesign and development of PHRs for the purpose of cancer and chronic disease management.

## Appendix

Multimedia Appendix 1 Screenshots of the Colorectal Cancer Survivor’s Personal Health Record (CRCS-PHR) user interface of specific features and functions.

## Abbreviations

CRC: colorectal cancer
PHR: personal health record
CRCS-PHR: Colorectal Cancer Survivor’s Personal Health Record
VA: Veterans Affairs
