# Supplementary material for: Usefulness and Usability of a Personal Health Record and Survivorship Care Plan for Colorectal Cancer Survivors: Survey Study
Source: JMIR Cancer. 2019 Aug 20;5(2):e10692. doi: 10.2196/10692 (PMC6719487; doi:10.2196/10692)

Screenshots of the Colorectal Cancer Survivor's Personal Health Record (CRCS-PHR)
User interface of specific features and functions

Figure 1: Relationships screenshot

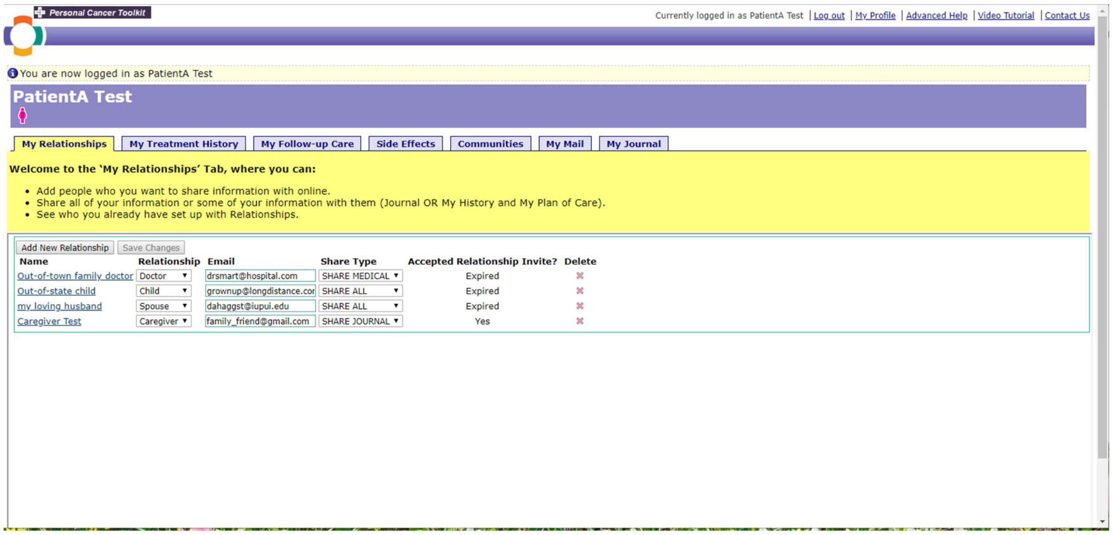

Figure 2: Treatment Summary screenshot

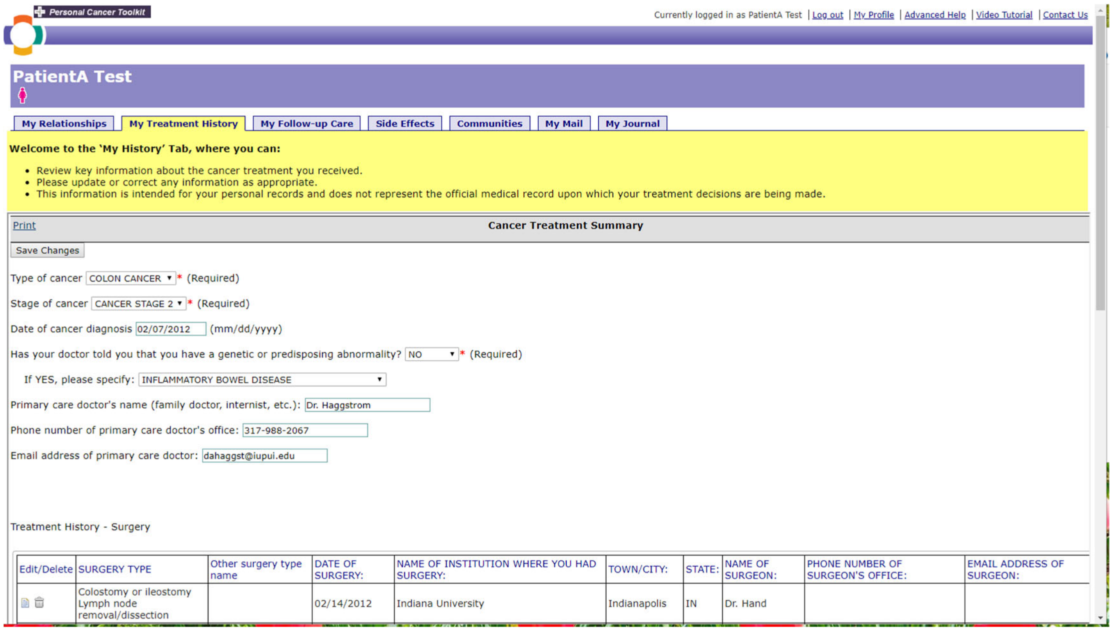

Figure 3: Surveillance Care screenshot

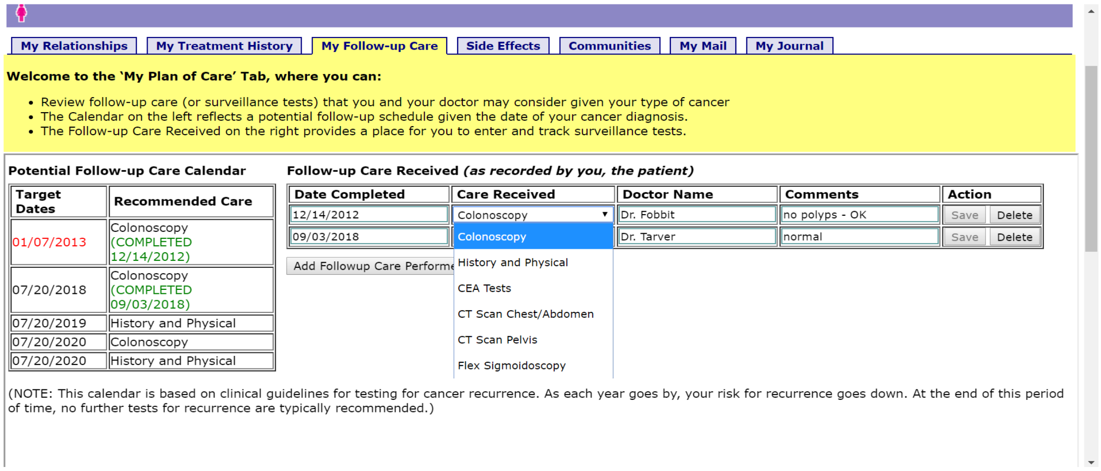

Figure 4: Side Effects screenshot

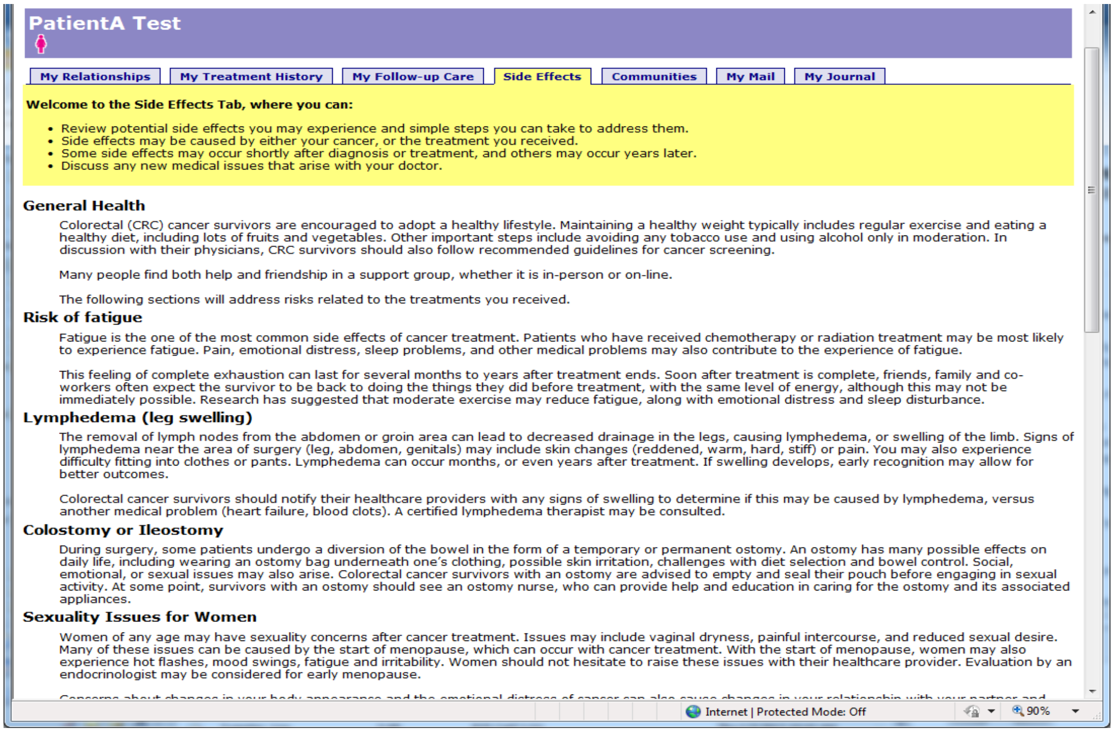

Figure 5: Community Resources screenshot

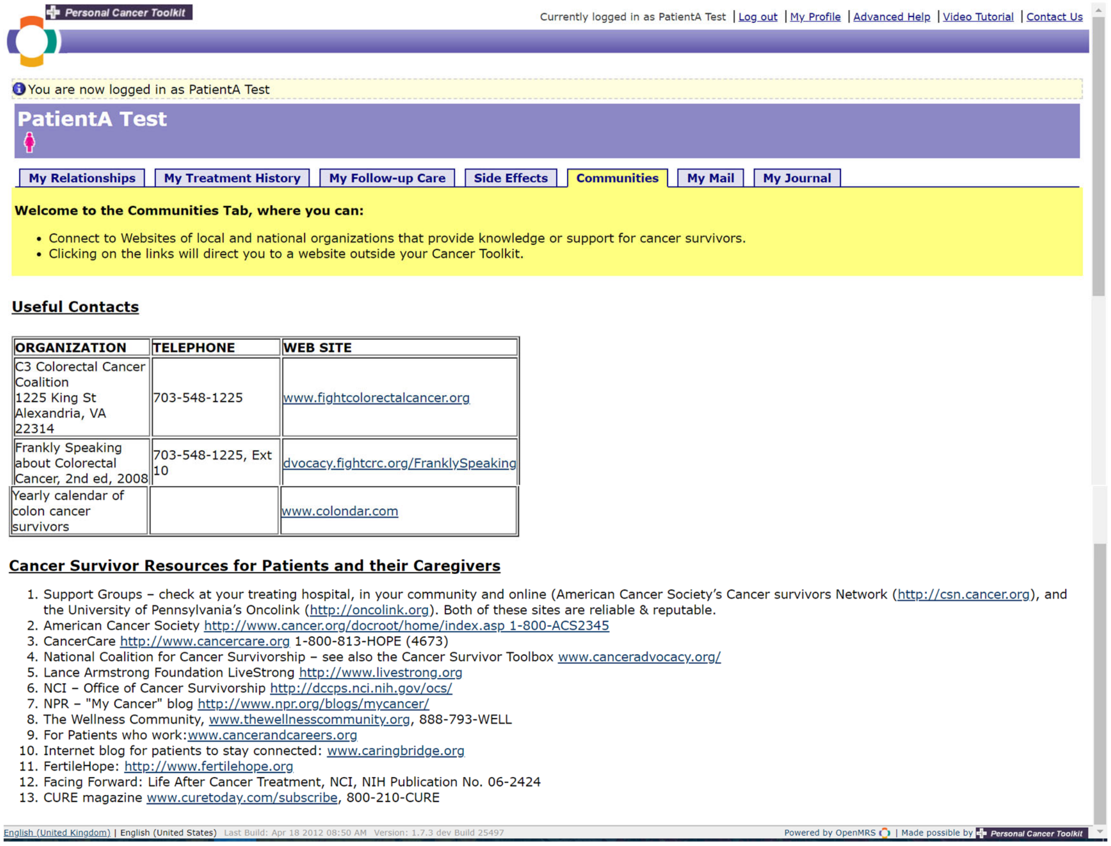

Personal Cancer Toolkit

Currently logged in as PatientA Test | Log out | My Profile | Advanced Help | Video Tutorial | Contact Us

You are now logged in as PatientA Test

PatientA Test

My Relationships | My Treatment History | My Follow-up Care | Side Effects | Communities | My Mail | My Journal

Welcome to the Communities Tab, where you can:

- Connect to Websites of local and national organizations that provide knowledge or support for cancer survivors.
- Clicking on the links will direct you to a website outside your Cancer Toolkit.

Useful Contacts

ORGANIZATION	TELEPHONE	WEB SITE
C3 Colorectal Cancer Coalition 1225 King St Alexandria, VA 22314	703-548-1225	www.fightcolorectalcancer.org
Frankly Speaking about Colorectal Cancer, 2nd ed, 2008	703-548-1225, Ext 10	dvocacy.fightcrc.org/FranklySpeaking
Yearly calendar of colon cancer survivors		www.colondar.com

Cancer Survivor Resources for Patients and their Caregivers

1. Support Groups – check at your treating hospital, in your community and online (American Cancer Society's Cancer survivors Network (http://csn.cancer.org), and the University of Pennsylvania's Oncolink (http://oncolink.org). Both of these sites are reliable & reputable.
2. American Cancer Society http://www.cancer.org/docroot/home/index.asp 1-800-ACS2345
3. CancerCare http://www.cancercare.org 1-800-813-HOPE (4673)
4. National Coalition for Cancer Survivorship – see also the Cancer Survivor Toolbox www.canceradvocacy.org/
5. Lance Armstrong Foundation LiveStrong http://www.livestrong.org
6. NCI – Office of Cancer Survivorship http://dccps.nci.nih.gov/ocs/
7. NPR – "My Cancer" blog http://www.npr.org/blogs/mycancer/
8. The Wellness Community, www.thewellnesscommunity.org, 888-793-WELL
9. For Patients who work:www.cancerandcareers.org
10. Internet blog for patients to stay connected: www.caringbridge.org
11. FertileHope: http://www.fertilehope.org
12. Facing Forward: Life After Cancer Treatment, NCI, NIH Publication No. 06-2424
13. CURE magazine www.curetoday.com/subscribe, 800-210-CURE

English (United Kingdom) | English (United States) Last Build: Apr 18 2012 08:50 AM Version: 1.7.3 dev Build 25497 Powered by OpenMRS | Made possible by Personal Cancer Toolkit

Figure 6: Secure Messaging screenshot

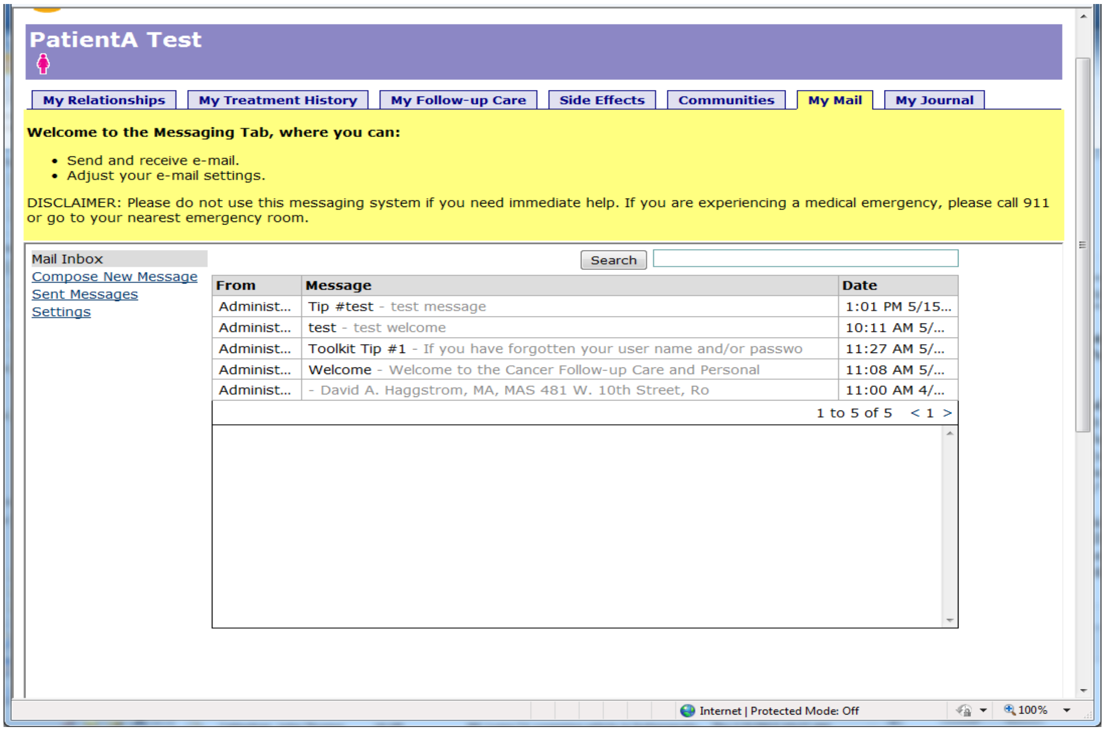

Figure 7: Journal screenshot

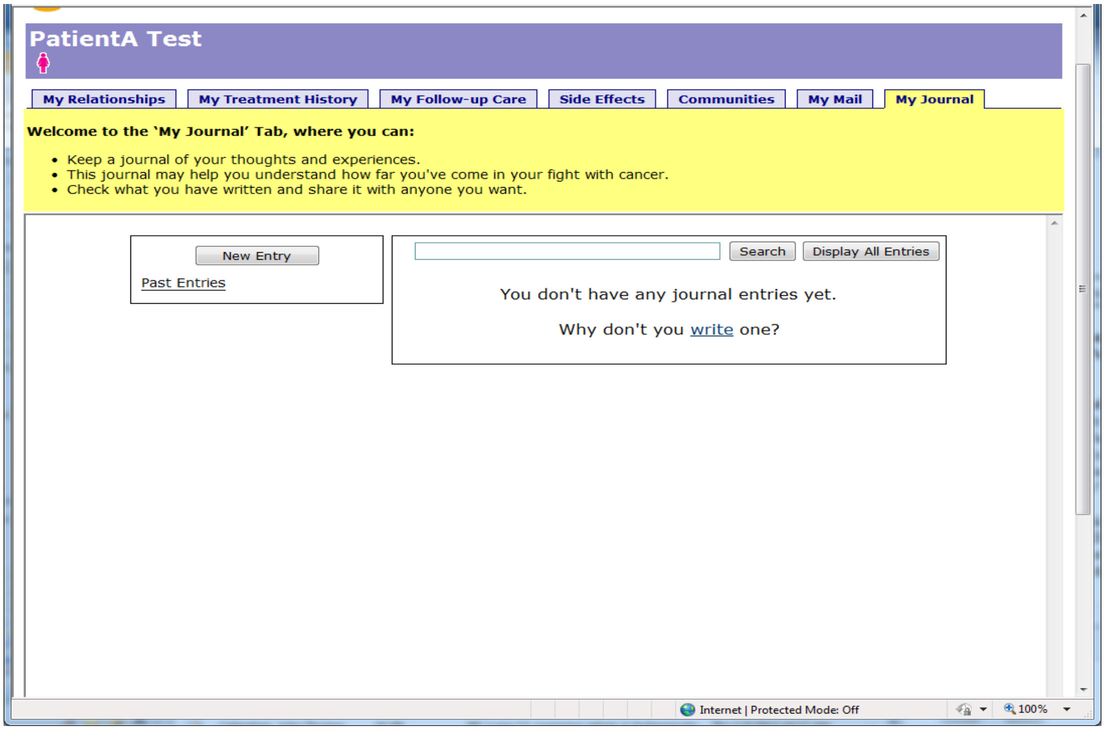

Supplement: Multimedia Appendix 1 [file cancer_v5i2e10692_app1.pdf]
